# 二代测序技术在骨髓增生异常肿瘤中的临床应用中国专家共识（2026年版）

**DOI:** 10.3760/cma.j.cn121090-20251106-00511

**Published:** 2026-01

**Authors:** 中华医学会血液学分会白血病淋巴瘤学组

## Abstract

骨髓增生异常肿瘤（MDS）是一组高度异质性髓系肿瘤，以血细胞减少、发育不良和高白血病转化风险为特征。二代测序（NGS）技术可高效检测基因突变，已被纳入MDS的诊断分型、预后评估及治疗决策体系。为进一步规范NGS技术在MDS临床实践中的应用，中华医学会血液学分会白血病淋巴瘤学组在整合MDS最新诊治进展、国内外相关指南/共识及国内专家建议的基础上，制定了本版专家共识，以推动NGS在我国MDS诊疗中的合理应用。

## Abstract (en)

Myelodysplastic neoplasms（MDS）are a group of highly heterogeneous myeloid malignancies characterized by cytopenia, dysplasia, and a high risk of leukemic transformation. Next-generation sequencing（NGS）technology enables efficient detection of gene mutations and has been incorporated into the diagnostic classification, prognostic assessment, and treatment decision-making systems for MDS. To further standardize the clinical application of NGS technology in MDS, Leukemia & Lymphoma Group, Chinese Society of Hematology, Chinese Medical Association have developed this expert consensus by integrating the latest advances in MDS diagnosis and treatment, relevant domestic and international guidelines/consensus recommendations, and expert opinions from China. The aim is to promote the rational use of NGS in the diagnosis and treatment of MDS in China.

骨髓增生异常肿瘤（myelodysplastic neoplasms，MDS），此前称骨髓增生异常综合征（myelodysplastic syndromes，MDS），是一组起源于造血干细胞和（或）祖细胞的高度异质性髓系肿瘤，表现为持续性的一系或多系血细胞减少、骨髓细胞发育异常和高风险向急性髓系白血病（acute myeloid leukemia，AML）转化[1]。二代测序（next-generation sequencing，NGS）技术具有高灵敏度和特异性，能一次对数十万条以上DNA序列片段进行测序分析，识别基因变异信息。基因突变作为克隆性造血的证据，被国内外指南推荐作为疑似MDS患者诊断的必检项目，同时也已引入MDS的诊断分型和预后危险度评估[2]–[4]。基因突变作为MDS治疗后可检测残留病（measurable residual disease，MRD）的监测也已有诸多报道。中西方MDS患者基因突变谱略有不同，国内MDS患者常见突变包括U2AF1、ASXL1、SF3B1、RUNX1、TET2和TP53等[5]–[6]。结合最新世界卫生组织（World Health Organization，WHO）和临床咨询委员会（clinical advisory committee，CAC）对MDS、潜能未定克隆性造血（clonal hematopoiesis of indeterminate potential，CHIP）和意义未明克隆性血细胞减少（clonal cytopenia of undetermined significance，CCUS）的诊断标准、美国国立综合癌症网络（National Comprehensive Cancer Network，NCCN）指南、欧洲白血病网络（European Leukemia Net，ELN）指南、最新临床研究报道及国内的专家共识等对NGS检测在MDS中的应用推荐，形成了此专家共识，以指导临床规范应用[2],[7]–[14]。

一、NGS在MDS临床实践中的应用

**推荐意见1**：基于MDS的WHO 2022和国际共识分类（International Consensus Classification，ICC）标准，NGS检测应作为MDS分型的关键依据。

（一）NGS在MDS诊断分型中的应用

1. TP53双等位基因失活的判定：WHO 2022分型建议将MDS分为伴遗传学异常的MDS和伴形态学异常的MDS两大类，前者的诊断优先级更高[3]。其中，MDS伴双等位TP53失活（MDS-biTP53）为具有最高诊断优先级的亚型，应予优先识别。根据WHO 2022标准，建议在满足以下任何1个条件时，诊断为MDS-biTP53：①有2个或2个以上不同的TP53突变位点；②至少1个TP53突变位点合并涉及染色体17p13.1（TP53位点）缺失的细胞遗传学异常；③至少1个TP53突变位点合并涉及染色体17p13.1（TP53位点）拷贝中性杂合性缺失（copy-neutral loss of heterozygosity，cnLOH）；④TP53突变的变异等位基因频率（variant allele frequency，VAF）大于49％[3],[15]–[16]。

MDS的ICC标准将原始细胞比例<10％且有TP53双等位基因突变的患者定义为MDS伴TP53突变（MDS with mutated TP53）[2]；对于原始细胞比例≥10％且合并TP53突变的MDS患者，无论TP53基因突变状态如何，均应定义为MDS/AML伴TP53突变（MDS/AML with mutated TP53）。与WHO标准相比，ICC标准要求：①TP53基因突变的VAF均需≥10％；②在cnLOH信息缺失的情况下，单个TP53突变合并任意复杂核型也可认为是TP53双等位基因突变（表1）。

**表1:** 世界卫生组织（WHO）和国际共识分类（ICC）标准MDS伴SF3B1突变和MDS伴TP53突变亚型的诊断要求对比

亚型	5^th^ WHO诊断标准^a^	ICC诊断标准^a^
MDS伴SF3B1突变	①原始细胞比例：BM<5％且PB<2％； ②无del（5q）、−7/del（7q）及复杂核型； ③合并SF3B1突变（无SF3B1突变的情况下，BM RS≥15％也可诊断）	①原始细胞比例BM<5％且PB<2％； ②无del（5q）、−7/del（7q）、3q26.2异常及复杂核型； ③SF3B1 VAF≥10％，不合并RUNX1突变
MDS伴双等位TP53失活	①BM和PB原始细胞<20％； ②TP53双等位基因失活^b^	–
MDS伴TP53突变	–	①BM和PB原始细胞<10％； ②多打击TP53突变^c^，或单个TP53突变（VAF>10％）合并复杂核型^d^
MDS/AML伴TP53突变	–	①BM和PB原始细胞10％～19％； ②TP53突变（VAF>10％，不要求突变状态）

**注** MDS：骨髓增生异常肿瘤；AML：急性髓系白血病；BM：骨髓；PB：外周血；RS：环形铁粒幼细胞；VAF：变异等位基因频率；–：不适用；^a^各诊断分型需满足相应所有条件；^b^WHO标准的TP53双等位基因失活定义详见正文描述；^c^ICC标准定义的多打击TP53突变：2个或以上TP53突变且VAF均>10％或单个TP53突变（VAF>10％）合并以下情况之一：①染色体del（17p）异常；②VAF>49％；③17p TP53位点拷贝中性杂合性缺失（cnLOH）；^d^在17p TP53位点cnLOH信息不可获知的情况下

TP53突变的MDS患者在按照WHO 2022与ICC标准分型时，可能出现诊断不一致的情况。国内队列研究表明[17]，对于符合WHO 2022定义的MDS-biTP53，但因TP53 VAF<10％而不满足ICC定义的“MDS或MDS/AML伴TP53突变”的患者，其修订的国际预后积分系统（revised international prognosis scoring system，IPSS-R）风险分层较低且总生存（OS）期更长[18]。鉴于此，对于此类诊断不一致的病例，建议优先采用ICC标准进行分型。

2. SF3B1突变相关分型的诊断规范：根据最新WHO诊断分型标准，对于合并SF3B1突变且骨髓原始细胞比例<5％的MDS患者，无论骨髓是否存在环形铁粒幼细胞（ring sideroblast，RS），均诊断为MDS伴SF3B1突变（MDS-SF3B1），但需除外染色体del（5q）、−7/del（7q）和复杂核型异常。同时WHO标准也认为，MDS患者无SF3B1突变但骨髓RS比例≥15％亦可诊断为MDS-SF3B1。而ICC标准则要求必须有SF3B1突变且VAF≥10％（表1）[2]。MDS-SF3B1是一组相对低危的MDS分子亚型，其临床异质性可受其他合并基因突变的影响。当合并RUNX1或EZH2突变时，MDS-SF3B1患者呈现更高危的临床表型，表现为更高的白血病转化风险和更短的OS期[19]。

3. MDS的分子分型：近期，Bernard等[20]基于基因组分析确定了DDX41、AML-like、TP53-CK、del（5q）、SF3B1、bi-TET2、der（1;7）、CCUS-like、−7/SETBP1、EZH2-ASXL1、IDH-STAG2、BCOR/L1、U2AF1^157^、U2AF1^34^、SRSF2、ZRSR2、mNOS和No-event 18个具有不同临床表现和预后的MDS分子亚型，其中一些分子亚型是目前已认定的，如TP53-CK、del（5q）、SF3B1。今后需要对更多分子亚型的机制、临床特征进一步研究，以期在治疗上获得突破。

（二）NGS在MDS治疗决策中的应用

**推荐意见2**：在拟定MDS治疗方案前评估基因突变状态，特别是SF3B1、TP53、IDH1/2、TET2等，以指导药物选择和疗效预判。

随着分子靶向药物在MDS中的不断应用，NGS在识别可靶向突变、预测药物疗效和规避治疗风险等方面的价值日益凸显。基因突变谱已成为制定个体化治疗策略的重要依据。

罗特西普（luspatercept）是一种转化生长因子-β（TGF-β）信号通路抑制剂，可解除Smad2/3介导的对红系分化的抑制作用，促进红细胞成熟。该药已获美国食品药品监督管理局（FDA）批准用于治疗红细胞输注依赖的低危MDS-RS成人患者。研究显示，SF3B1突变阳性的MDS患者对罗特西普治疗反应率更高，在PACE-MDS研究中，其红系血液学改善（HI-E）率达77％，显著高于突变阴性患者（40％），且多因素分析结果证实SF3B1突变阳性是国际工作组（International Working Group，IWG）HI-E的预后良好因素[21]。因此，对于低危MDS伴SF3B1突变，尤其是MDS-RS患者，可考虑优先使用罗特西普治疗。

来那度胺是MDS伴5号染色体长臂缺失［MDS-del（5q）］患者的一线治疗药物，可通过E3泛素连接酶底物受体蛋白（CRBN）介导降解酪氨酸蛋白激酶1α1（CSNK1A1）和Ikaros家族锌指蛋白1/3（IKZF1/3），诱导肿瘤克隆凋亡[22]。TP53突变在MDS-del（5q）中的发生率显著高于其他MDS亚型，且突变率在白血病转化阶段进一步升高[23]。TP53突变的MDS-del（5q）患者对来那度胺疗效欠佳，且来那度胺可能导致TP53突变克隆选择性扩增，增加白血病转化风险[24]–[25]。因此，建议在MDS-del（5q）患者接受来那度胺治疗前评估TP53突变状态。对于合并TP53突变者，应谨慎使用来那度胺，并加强治疗随访与复发监测。

IDH1突变为部分MDS患者提供了靶向治疗新选择。IDH1抑制剂艾伏尼布（ivosidenib）在复发/难治性MDS患者中展现出显著疗效，Ⅰ期研究的总缓解率（ORR）达83.3％，部分患者摆脱了输血依赖，中位OS期达35.7个月。基于该研究，FDA于2023年10月批准其用于治疗IDH1突变的复发/难治性MDS。研究亦提示，应用艾伏尼布未获得治疗反应的患者IDH1突变中位VAF为3％，提示IDH1突变负荷较低时艾伏尼布可能无法清除优势克隆[26]。因此，推荐IDH1突变且VAF>5％的复发/难治性MDS患者使用艾伏尼布治疗。

正在进行的Ⅱ期IDIOME临床试验进一步探讨了艾伏尼布单药或联合去甲基化药物（hypomethylating agents，HMA）治疗复发难治IDH1突变MDS患者的安全性和疗效[27]。此外，新一代IDH1抑制剂olutasidenib、IDH2抑制剂恩西地平及p53激活剂Eprenetapopt等靶向药物也在MDS患者中进行临床试验验证，其疗效与安全性有待进一步研究[28]–[30]。对于IDH1/2突变或TP53突变的MDS患者，应密切关注新型靶向药物的临床研究进展，并在合适条件下参加临床试验。

（三）NGS技术在MDS预后判断中的应用

**推荐意见3**：推荐在MDS初诊评估时使用包含基因突变的国际预后积分系统（Molecular IPSS，IPSS-M），以实现精准预后分层。

2022年MDS预后国际工作组（International Working Group for Prognosis in MDS，IWG-PM）提出了IPSS-M预后积分系统，在传统临床参数基础上引入了31个突变基因，是目前国际上最系统的MDS分子风险模型。IPSS-M基于连续风险指数，综合评估以下因素：血液学参数、骨髓原始细胞比例、细胞遗传学风险分组、16个主要预后相关突变基因（ASXL1、CBL、DNMT3A、ETV6、EZH2、FLT3、IDH2、KRAS、MLLPTD、NPM1、NRAS、RUNX1、SF3B1、SRSF2、multi-hit TP53和U2AF1），以及15个次要突变基因（BCOR、BCORL1、CEBPA、ETNK1、GATA2、GNB1、IDH1、NF1、PHF6、PPM1D、PRPF8、PTPN11、SETBP1、STAG2和WT1）中突变基因的数量。通过在线网页（https://mds-risk-model.com/）可实现简单运算，并提供个性化评分，对OS、白血病转化风险及无白血病生存期进行预测[4]。多项研究证实，IPSS-M对上述所有临床终点的预测效力（OS、白血病转化风险、无白血病生存期）均优于IPSS-R[18]。

尽管IPSS-M在精度上取得显著提升，但其在临床实践中的应用仍面临挑战。首先，该系统包含的基因数量较多，可能增加检测标准化难度。其次，研究表明，IPSS-M模型的准确性高度依赖于输入数据的完整性。对于资源有限、无法开展全基因组或31个基因完整检测的中心，建议优先涵盖主要预后相关基因，此时IPSS-M模型的预测准确率尚可维持在70％～80％。若检测覆盖的主要预后突变基因不足10个，IPSS-M的预测效能将显著降低。在此情况下，不推荐强制使用IPSS-M评分，以免因核心数据缺失导致风险误判，误导临床决策[31]。此外，国内MDS患者与国外IWG-PM队列存在以下差异：中位发病年龄更低，血细胞减少程度更严重、骨髓原始细胞增多及不良染色体核型比例更高。国内研究队列显示，IPSS-M在年龄>60岁的MDS患者中预测效力更强，提示IPSS-M的风险评估需整合年龄等患者相关因素以优化预测效能[32]。

正常核型MDS（NK-MDS）患者中，TP53突变的发生率较低，不是独立的危险因素。而CEBPA、U2AF1和RUNX1突变在NK-MDS中具有独立预后意义[33]–[35]。整合上述基因状态与传统临床参数如年龄、骨髓原始细胞比例、血红蛋白水平、血小板计数构建的NK-PSS-M评分系统可实现对NK-MDS患者的有效分层，其预测性能与IPSS-M相当。在资源受限无法开展完整IPSS-M评估时，可作为对NK-MDS预后评估的替代方案[35]。

（四）NGS技术在MDS克隆演变监测中的应用

**推荐意见4**：可将NGS用于MDS患者的动态随访，监测基因突变谱变化，以识别克隆演变、疾病进展及疗效监测。

1. MDS患者克隆演变的分子特征与监测重点：

（1）信号通路相关的基因突变（NRAS、KRAS、CBL、NF1、PTPN11、FLT3、KIT、IDH1、IDH2）与细胞增殖相关，此类突变常新发于MDS进展为AML的过程中，对该类基因进行密切的分子监测可早期预测MDS白血病转化风险[36]。

（2）TP53突变的患者，在向AML转化的过程中则更常出现TP53克隆扩增、VAF升高，而非新发的基因突变[37]，应重点监测TP53 VAF变化。

（3）DDX41胚系突变的MDS患者往往表现为骨髓低增生、低肿瘤负荷及低突变频率。此类患者AML转化的机制尚不清晰，可能与年龄相关的造血压力相关，AML转化的过程中不常出现新发基因突变[38]，建议将克隆负荷及造血功能变化作为监测重点。

2. NGS监测治疗相关的克隆演变：NGS技术可通过超高深度测序检测到低至0.01％的肿瘤细胞残留，为传统疗效评价标准提供分子层面的补充信息。治疗后若观察到关键突变克隆（如TP53、RUNX1、SRSF2、DDX41等）清除，提示疗效较好。经治疗完全缓解的患者也可仍然存在较大的克隆（如DNMT3A、TET2），提示仍存在克隆性造血状态。此外，治疗过程中出现新突变（如RUNX1、TET2、CBL、DDX41、PPM1D和TP53等）往往提示复发风险升高、缓解深度不足[39]–[40]。然而，利用NGS监测MDS MRD存在费用较高、低频突变的临床意义尚不明确、易引发假阳性或过度解读、标准化不足、质控流程不明确及临床验证滞后等局限，需要不断完善和规范。

3. NGS在接受异基因造血干细胞移植患者中的指导作用：NGS可在移植前识别携带不良预后相关突变的患者。移植前检测到TP53突变和信号通路基因（NRAS、KRAS、CBL、NF1、RIT1、PTPN11、FLT3、KIT）突变与移植后早期复发和较短的生存期相关，TP53突变患者即便在缓解状态下接受移植，也表现为较短的生存期和较高的复发风险[41]。此外，如检测到DDX41、CEBPA、RUNX1、GATA2等胚系致病变异，应进一步通过家系验证评估供者适配性并进行遗传咨询。

移植后NGS监测有助于早期识别分子复发。移植后第100天内若检测到VAF≥0.5％的疾病相关突变，提示复发风险显著增加，患者OS期和无进展生存期显著缩短[42]。研究表明，分子学复发和血液学复发之间的平均间隔时间为71 d，是进行抢先治疗的最佳窗口期[43]。

4. NGS监测时点：建议在MDS初次诊断、充分治疗后疗效判断、治疗过程中出现疗效丧失、造血干细胞移植前评估、移植后每3～6个月以及疾病进展时进行NGS检测，以调整治疗策略、监测疾病进展和评估预后。对于较低危MDS患者，在随访过程中建议每年至少进行1次NGS检测。

（五）NGS用于遗传易感MDS的筛查

**推荐意见5**：对以下人群进行肿瘤易感基因筛查：①所有≤50岁的患者，但值得注意的是，携带DDX41胚系基因突变的患者常>50岁；②低增生性MDS；③新诊断的MDS合并其他肿瘤；④携带胚系突变患者，其亲缘造血干细胞供者；⑤任何年龄的临床疑似遗传易感性综合征，早发性或多重癌症、对化疗或放疗表现出过度毒性、干细胞动员不良、不明原因的血细胞减少或大红细胞增多症、肺纤维化或肝纤维化、合并先天免疫缺陷、实验室检查结果提示免疫缺陷或免疫失调。

MDS存在遗传易感性，基因胚系突变增加肿瘤易感性，合并体细胞基因突变二次打击促进肿瘤发生[44]–[45]。MDS患者携带胚系突变的频率为5％～10％，在年轻MDS患者中检出频率更高[46]。DDX41是目前已知MDS中最常见的胚系易感基因[47]，ICC[2]及WHO 2022[3]分类标准均将DDX41相关髓系肿瘤列为独立亚型。北欧髓系肿瘤胚系易感性工作组在此基础上建议所有髓系肿瘤基因筛查纳入DDX41分析，并进行基线血液学评估和专业遗传咨询[48]。根据最新NCCN指南[14]，部分人群需进行肿瘤易感基因筛查，形成推荐意见5。

二、NGS检测推荐标准流程

（一）样本采集与处理

NGS检测前应征得患者知情同意。患者应充分了解基因检测的可能获益及局限性。通常采集骨髓液，采集量一般为2～3 ml，采用专用抗凝采血管，如EDTA管，样本采集后应尽快4 °C冷藏运输到实验室进行检测。对于因骨髓纤维化等导致骨髓干抽无法获取足够骨髓液的患者，可采集外周血。建议采用含细胞稳定剂的游离DNA专用抗凝管，如Streck管，外周血一般为10 ml，常温条件下可稳定保存3～7 d，通过外周血进行NGS检测[49]–[52]，检测后保存剩余样本。

（二）样本检测要点

基于NGS检测的靶向测序策略主要分为2种，为基于杂交捕获和基于扩增子的靶向测序。前者通过捕获探针与基因组中特定序列杂交，获得目标区域序列，后者基于多重PCR扩增完成目标序列富集。两种测序策略各有优劣，临床医师应根据具体的基因检测组合大小、基因数量和时效性选择。前者建库时间长，样本目标序列覆盖均一性高，除常规可检测点突变、小片段插入和缺失外，可检测未知基因重排；后者建库时间短，扩增均一性需在引物设计时优化，适用于基因数量少、明确的热点区域检测。无论采用何种测序策略，均应符合明确的合格文库标准[53]–[54]。

（三）基因检测组合选择

关于基因检测组合的选择，应由临床专家和实验室专家共同决定，相关依据参考ELN、NCCN、WHO 2022、ICC指南对MDS、CHIP、CCUS及胚系易感倾向髓系肿瘤的诊断标准，IPSS-M预后积分系统包含基因组套以及中华医学会等机构指南或共识[2]–[4],[7],[14]。建议检测的体细胞突变和易感的胚系突变基因见表2。

**表2:** 骨髓增生异常肿瘤相关突变基因列表

基因类型	基因
体细胞基因	TET2、DNMT3A、ASXL1、EZH2、SF3B1、SRSF2、U2AF1、ZRSR2、RUNX1、TP53、STAG2、NRAS、CBL、NF1、JAK2、CALR、MPL、ETV6、GATA2、DDX41、IDH1、IDH2、SETBP1、PHF6、BCOR、FLT3、WT1、GNAS、ZBTB33、NPM1、STAT3、PPM1D、UBA1、KMT2A、KRAS、BCORL1、CEBPA、ETNK1、GNB1、PRPF8、PTPN11、CTCF、BRCC3
胚系基因	CEBPA、DDX41、ANKRD26、ETV6、GATA2、RUNX1、SAMD9、SAMD9L、SRP72、TP53

筛查突变类型应包括单核苷酸变异（single nucleotide variation，SNV）、小片段插入缺失（insertion and deletion，Indel）、FLT3内部串联重复（FLT3-ITD）和KMT2A部分串联重复（KMT2A-PTD）。关于检测区域的选择，应考虑临床诊疗指南、专家共识、权威数据库及实验室自建数据库。具体指导原则如下：①有明确基因突变热点区域，应优先纳入，如TET2、DNMT3A、SF3B1、SRSF2、U2AF1；②无明确基因突变热点区域，应纳入全部外显子，如TP53、EZH2、ASXL1、RUNX1、BCOR、NF1；③影响蛋白功能的剪切位点区域，应纳入剪切位点至少±5 bp范围；④非翻译区突变基因，应纳入相应区域，如ANKRD26、GATA2。

（四）NGS检测报告和结果解读

基因检测报告解读的呈现应由临床专家和实验室专家共同制定，突变位点的临床意义解读应简明清晰、明确易懂。检测报告应明确患者基本信息、样本信息、实验室测序平台与测序策略、测序深度、突变基因检测内容、质控信息，并明确VAF的检测下限（limit of detection，LOD），建议报告VAF LOD≥2％。报告首页应汇总所有检测阳性信息。突变位点的检测结果应纳入突变基因、染色体定位、转录本号、外显子、核苷酸改变、氨基酸改变、突变频率及拷贝数变异（copy number variant，CNV）等条目信息，位点临床意义的解读应参考国内外MDS诊疗指南、权威数据库（如COSMIC、ClinVar、HGMD、TCGA）和重要文献，并附上来源。

关于突变位点的分级，参照《二代测序技术在血液肿瘤中的应用中国专家共识（2018年版）》[7]将变异等级分为三类：一级变异，具有明确临床意义的突变；二级变异，具有潜在临床意义的突变；三级变异，临床意义未明的突变。

三、结语

随着对MDS认知的不断加深、NGS成本的降低，合理与规范地应用NGS检测将为MDS的诊断及鉴别诊断、分型、预后分层及治疗提供重要依据。未来需要开展更多的研究，进一步探索MDS分子生物学异常的价值。临床实践中，应组建分子肿瘤专家委员会，纳入临床医师、病理科医师、检验科医师、分子生物学专家与生物信息学专家等多专业领域专家，进行多学科合作。通过对患者临床诊疗信息、基因检测结果与治疗方案的整合实现患者的全病程管理，提升患者临床获益。
